# Environmental metabarcoding reveals heterogeneous drivers of microbial eukaryote diversity in contrasting estuarine ecosystems

**DOI:** 10.1038/ismej.2014.213

**Published:** 2014-11-25

**Authors:** Delphine Lallias, Jan G Hiddink, Vera G Fonseca, John M Gaspar, Way Sung, Simon P Neill, Natalie Barnes, Tim Ferrero, Neil Hall, P John D Lambshead, Margaret Packer, W Kelley Thomas, Simon Creer

**Affiliations:** 1 Molecular Ecology and Fisheries Genetics Laboratory, School of Biological Sciences, Environment Centre Wales, Bangor University, Bangor, UK; 2 School of Ocean Sciences, Bangor University, Anglesey, UK; 3 Zoological Research Museum Alexander Koenig (ZFMK), Centre for Molecular Biodiversity Research, Bonn, Germany; 4 Hubbard Center for Genome Studies, University of New Hampshire, Durham, NH, USA; 5 Department of Biology, Indiana University, Bloomington, IN, USA; 6 The Natural History Museum, Zoology Department, London, UK; 7 Advanced Genomics Facility, Institute of Integrative Biology, University of Liverpool, Liverpool, UK; 8 School of Ocean & Earth Science, University of Southampton, National Oceanography Centre, Southampton, UK

## Abstract

Assessing how natural environmental drivers affect biodiversity underpins our understanding of the relationships between complex biotic and ecological factors in natural ecosystems. Of all ecosystems, anthropogenically important estuaries represent a ‘melting pot' of environmental stressors, typified by extreme salinity variations and associated biological complexity. Although existing models attempt to predict macroorganismal diversity over estuarine salinity gradients, attempts to model microbial biodiversity are limited for eukaryotes. Although diatoms commonly feature as bioindicator species, additional microbial eukaryotes represent a huge resource for assessing ecosystem health. Of these, meiofaunal communities may represent the optimal compromise between functional diversity that can be assessed using morphology and phenotype–environment interactions as compared with smaller life fractions. Here, using 454 Roche sequencing of the 18S nSSU barcode we investigate which of the local natural drivers are most strongly associated with microbial metazoan and sampled protist diversity across the full salinity gradient of the estuarine ecosystem. In order to investigate potential variation at the ecosystem scale, we compare two geographically proximate estuaries (Thames and Mersey, UK) with contrasting histories of anthropogenic stress. The data show that although community turnover is likely to be predictable, taxa are likely to respond to different environmental drivers and, in particular, hydrodynamics, salinity range and granulometry, according to varied life-history characteristics. At the ecosystem level, communities exhibited patterns of estuary-specific similarity within different salinity range habitats, highlighting the environmental sequencing biomonitoring potential of meiofauna, dispersal effects or both.

## Introduction

Biodiversity contributes to ecosystem stability, resilience, function (Loreau *et al.,* 2001; Wardle *et al.,* 2004) and the continued provision of ecosystem services (Schröter *et al.,* 2005), but is subject to a range of natural and anthropogenic forces. By understanding the natural processes that affect different facets of biodiversity, the effect of environmental change (Christensen *et al.,* 2007) and stressors (Chariton *et al.,* 2010) can then be disentangled from natural ecological processes in terrestrial and aquatic environments.

Estuaries are often centres of human habitation (Basset *et al.,* 2012) and are therefore the focus of intensive and costly biomonitoring programmes (Baird and Hajibabaei, 2012) designed for assessing ecosystem health (Rosenberg *et al.,* 2004). Estuaries are transitional habitats (Elliott and Whitfield, 2011) that are typified by diverse hydrodynamic flows, diurnal tidal cycles and radically different biological communities as the result of species adaptations to freshwater, marine and intermediate salinity regimes (Attrill, 2002; Attrill and Rundle, 2002). In attempts to make predictions about the biodiversity of a typical estuary, many studies refer to the somewhat arbitrary Remane model (based upon the Baltic Sea) that models species richness along a salinity continuum (Whitfield *et al.,* 2012). The Remane model and recent modifications refined by Whitfield *et al.* (2012) both predict the lowest species diversity between the oligohaline (0.5–5.0 parts per thousand (p.p.t.)) and mesohaline (5–18 p.p.t.) zones, with peaks in euhaline (30–40 p.p.t.) and freshwater areas. Alternatively, Attrill (2002) proposes a different model that predicts a reduction of species richness with increasing variation of local salinity.

Although diatoms and macroinvertebrates commonly feature as focal bioindicator species, the diversity of additional small organisms represent a potentially huge resource for assessing ecosystem health (Baird and Hajibabaei, 2012). Of the different organisms inhabiting benthic sediments, the meiofauna (animals between 45 and 500 μm) (Giere, 2009) might represent the optimal compromise for biomonitoring potential between diversity in life-history characteristics, taxonomic tractability and temporal stability as compared with bacteria and smaller protist groups. Approximately 60% of animal phyla have meiofaunal representatives with communities being numerically dominated by nematodes (Giere, 2009). However, the potential of using such small organisms for assessing ecosystem health, as demonstrated by Chariton *et al.* (2010), has never been fully realised because of taxonomic and logistical limitations (Creer *et al.,* 2010).

The synergies afforded by the advent of ‘second-generation sequencing' platforms (Glenn, 2011) and necessary taxonomic database reference libraries (Pruesse *et al.,* 2007) present an unprecedented opportunity to assess the biodiversity of previously intractable communities. In such studies, the DNA from entire communities are extracted *en masse*, and barcodes are amplified via PCR, sequenced, grouped into genetically similar units (operational taxonomic unit (OTU) clustering) and assigned to the appropriate taxon using reference databases. Such *en masse* biodiversity assessments of microbial communities from environmental samples (Bik *et al.,* 2012) have been termed metabarcoding (Taberlet *et al.,* 2012b), offering the potential for an analytical framework referred to as Biomonitoring 2.0 (Baird and Hajibabaei, 2012). This type of monitoring requires an effective understanding of the relationships between communities quantified using monitoring and ecosystem-related effects. For the meiofaunal size fraction, many natural drivers of biodiversity have been proposed, but of these, sediment granulometry (Giere, 2009), salinity range (Attrill, 2002), hydrodynamic flows (Heip *et al.,* 1985) and top-down processes such as bioturbation (Graf, 1992) have been hypothesised to strongly affect community diversity.

In this study, we adopt a metabarcoding approach to investigate whether sediment composition, hydrodynamics, salinity range or levels of bioturbation have the largest effects on the biodiversity of numerous phyla at the estuarine ecosystem scale. In order to investigate potential variance at the ecosystem scale, we compare two contrasting UK estuarine ecosystems: Thames and Mersey. Thames is considered a ‘recovered' estuary according to ecotoxicological history (Power *et al.,* 1999; Matthiessen and Law, 2002), whereas Mersey, recognised in the past as one of the most polluted estuaries in Europe (NRA, 1995), is now improving because of regulation and environmental campaigns (Struthers, 1997). Such histories therefore are predicted to skew the relationships between the biodiversity of different phyla and environmental drivers according to ecosystem-specific effects.

## Materials and methods

### Sample collection and community decantation

**Figure 1:**
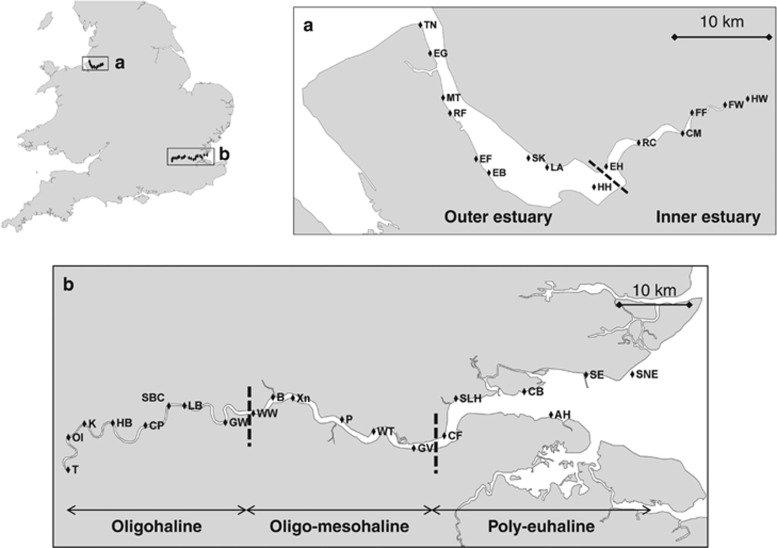
Map of the sampling locations. (**a**) Mersey estuary. CM, Cuerdley Marsh; EB, Ellesmere Bank; EF, Eastham Ferry; EG, Egremont; EH, East Hale; FF, Fiddlers Ferry; FW, Forest Way; HH, Hale Head Shore; HW, Howley Weir; LA, Liverpool Airport; MT, Mersey Tunnel; RC, Runcorn; RF, Rock Ferry; SK, Speke; TN, The Narrows. (**b**) Thames estuary. AH, Allhallows; B, Beckton; CB, Cavney Island; CF, Coalhouse Fort; CP, Cadogan Pier; GV, Gravesend; GW, Greenwich; HB, Hammersmith Bridge; K, Kew; LB, London Bridge; OI, Old Isleworth; P, Purfleet; SBC, South Bank Centre; SE, Southend-on-Sea; SLH, Stanford Le Hope; SNE, Shoebury Ness; T, Teddington; WT, West Thurrock; WW, Woolwich; XN, Crossness. Salinity zones have been named according to the Venice salinity classification system: oligohaline (0.5–5‰), mesohaline (5–18‰), polyhaline (18–30‰), euhaline (30–40‰) and hyperhaline (>40‰).

In all, 104 benthic samples were collected from Thames (20 sampling stations) and Mersey (15 sampling stations) estuaries (UK) in June–July 2008. For both estuaries, benthic communities were sampled at the low-tide mark, accessed either on foot (Thames) or by boat (Mersey) (Supplementary Table S1 and Figure 1). At each station, 3 sediment core samples were collected using Perspex tubes (4.4 cm in diameter, 10 cm deep, ∼10 m apart) for metabarcoding analysis of meiofauna, each being stored in 500 ml of DESS (20% dimethyl sulphoxide and 0.25 M disodium EDTA, saturated with NaCl, pH 8.0, Yoder *et al.,* 2006). A fourth core sample was collected for granulometric analysis. In the laboratory, the meiofaunal size fraction and organisms up to 1 mm in size were mechanically separated from the sediment and immobilised on a 45 μm filter before separation from fine silt using repetitive centrifugations in 1.16 specific gravity LUDOX TM-40 solution (Sigma-Aldrich Company Ltd., Gillingham, UK) (Fonseca *et al.,* 2011). Following this step, each sample was retained on a distinct mesh sieve that was then folded, sliced, placed in a 15 ml Falcon tube and kept at −80 °C until DNA extraction. After overnight lysis at 55 °C, community DNA was extracted with the QIAamp DNA Blood Maxi (Qiagen, Manchester, UK) according to identical protocols set out in Fonseca *et al.* (2011). The highly conservative metabarcoding primers SSU_F_04 and SSU_R_22 (Blaxter *et al.,* 1998; Fonseca *et al.,* 2010) were used as they amplify broadly throughout meiofaunal organisms (in addition to protists and fungi) and they flank the most variable (in meiofaunal taxa) ∼450 bp nSSU gene region. The nSSU gene region was then PCR amplified in triplicates from community DNA using *Pfu* DNA polymerase (Promega, Southampton, UK) and forward and reverse MID-tagged fusion primers; visualised by gel electrophoresis and purified using the QIAquick Gel Extraction Kit (Qiagen); quantified on an Agilent Bioanalyser 2100 (Agilent Technologies, Stockport, UK) and pooled in equimolar quantities. The purified amplicons pools were then sequenced in a single direction (A-Amplicon) on four half plates using the 454 Roche GSFLX (454 Life Sciences, Roche Applied Science, Branford, CT, USA) sequencing platform at Liverpool University's Centre for Genomic Research (Liverpool, UK). All protocols were identical to those presented in Fonseca *et al.* (2010).

Raw sequence reads were filtered and denoised using FlowClus (Gaspar and Thomas, submitted, freely available at GitHub (jsh58/FlowClus)). Criteria used for the filtering step were: minimum sequence length 150 bp; maximum sequence length 500 bp; truncate reads before first N; truncate before a window of 25 bp whose average quality score is <20; truncate before a set of four flows whose values are <0.40 (criteria recommended by Reeder and Knight, 2010). The denoising step corrects pyrosequencing errors by clustering the flowgrams and a constant denoising value of 0.50 was used. Then, the data were analysed using the QIIME pipeline (Caporaso *et al.,* 2010): (1) chimeras were removed using UCHIME (Edgar *et al.,* 2011), with the abundance information generated by FlowClus; (2) OTUs were clustered at 96% sequence similarity using UCLUST (Edgar, 2010), as 96% sequence similarity has most closely emulated species richness via the analysis of control nematode communities using nSSU (Fonseca *et al.,* 2010); (3) a representative sequence was picked for each OTU; (4) taxonomy was assigned using the Silva 111 database (Pruesse *et al.,* 2007); and (5) an OTU table was generated. For direct ecological comparisons among samples that have different coverages (that is, number of reads), the percentage of reads in each sample was used instead of read counts and downstream analyses were focused on meiofauna and dominant protist groups occupying shallow sediment habitats. Raw sequence reads were additionally analysed using the OCTUPUS pipeline (Fonseca *et al.,* 2010, available at http://octupus.sourceforge.net/) and OTUs annotated against the downloaded NCBI (National Center for Biotechnology Information) nucleotide database using the raw data set and also a rarefied data set (1102 randomly picked sequences from each sample).

### Environmental data and macrofaunal biodiversity

Key environmental parameters, known to influence the distribution of meiofaunal communities, were measured or modelled below.

Salinity range (the difference between mean low-tide salinity and mean high-tide salinity) data (Attrill, 2002) were inferred from the Environment Agency (EA, United Kingdom) salinity data, as explained below. For Thames, mean salinity at each sampling site was calculated from the EA spot sampling data (along the estuary from Teddington to Barrow No. 7 Buoy). Mean salinity range was then inferred from the equation established by Attrill (2002) between mean salinity and salinity range. In the Mersey estuary, salinity range at the EA sampling stations was inferred from monthly EA salinity data. We constructed a model of the mean salinity range against the distance from Howley Weir (from Monks Hall to Seacombe Ferry: 4.50 and 41.67 km from Howley Weir, respectively) and from that relationship, salinity range was inferred at each of our 15 sampling sites.

Longitudinal variations in the time-varying free surface and current velocity were calculated using a one-dimensional sectional-averaged model (Neill *et al.,* 2009) that discretely solves the continuity equation


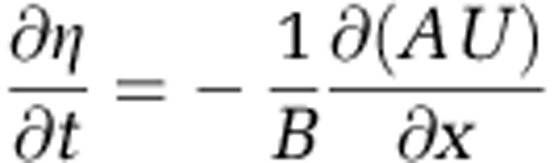


and the momentum equation


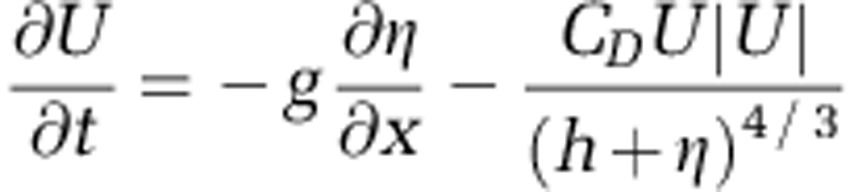


where *η* is the variation of the free surface from mean sea level, *B* is the channel width, *A* is the cross-sectional area of the channel, *U* is the depth-averaged velocity, *C*_*D*_ is the bottom friction coefficient, *h* is the mean water depth, *ρ* is water density, *δx* is the longitudinal grid spacing and *g* is gravitational acceleration. Cross-sectional areas for each river/estuary system were digitised from Admiralty Charts to a longitudinal grid spacing of *δx*=500 m, and the amplitudes and phases of four tidal constituents (including the dominant semidiurnal constituents) were obtained from an analysis of tide gauge data to provide model boundary conditions. The models were validated against tide gauge data along the length of each estuarine system. The models were run for the duration of a spring–neap cycle (∼2 weeks), and statistics calculated on tidal range, velocities and bed shear stress at each of the sampling locations.

Two methods were used for the sediment particle size analysis, depending on whether the sample was composed of predominantly fine or coarse material. Nine of the coarser samples were mechanically sieved and for the 26 finer samples, particle size analysis was carried out using a Malvern Mastersizer 2000 (Kenny and Sotheran, 2013). In contrast to mechanical sieving, the Mastersizer determines particle size distribution by volume. However, if we assume that the density of individual particles is constant, the results are directly comparable. Plots of cumulative particle size distributions were used to calculate the values of the particle diameter at 50% (median grain size D50) and 10% (D10) in the cumulative distribution of grain sizes, and to calculate the percentage of material contained within each size class.

Macrofaunal invertebrates were sampled at each station in the two estuaries by taking five 15 cm diameter cores to a depth of 10 cm. The cores were pooled and sieved over 1 mm and preserved in 4% formalin. All invertebrates were identified to the highest possible taxonomic resolution and their wet weight was measured after blotting. Macrofaunal species richness, abundance and biomass were calculated. In the Thames estuary, no cores could be taken at London Bridge because of the rocky substrate, and therefore only qualitative data were recorded at this site; no cores could be taken at Cadogan Pier and Kew because of a high fraction of large pebbles in the sediment.

### Statistical analyses to identify the drivers of meiofaunal diversity

Independent samples *t*-tests (including Levene's test for equality of variances) were performed to test whether there were significant differences in sample OTU richness between the Thames and Mersey estuaries. Multivariate analyses were performed to investigate the similarity of meiofaunal communities along the salinity gradient and to identify environmental drivers of meiofaunal assemblages. Before multivariate analysis in PRIMER v6 (Clarke and Gorley, 2006), the biotic data of meiofaunal OTUs were transformed into a presence/absence matrix of each OTU in each sample (only for OTUs annotated as Nematoda, Platyhelminthes (Turbellaria), Arthropoda, Mollusca, Annelida, Gastrotricha, Tardigrada, Kinorhyncha, Rotifera, Cnidaria and Bryozoa). Analyses described below were performed for each estuary and for the two estuaries combined. First, the similarity between meiofaunal assemblages along the salinity gradient was analysed by cluster analysis using group average clustering and ordination by non-metric multidimensional scaling, using the Sørensen's similarity coefficient (Clarke, 1993). The SIMPROF procedure (or ‘similarity profile' permutation tests) was applied to identify genuine clusters of samples, that is, samples that were not significantly differentiated from each other were grouped in the same cluster. Second, a BIOENV (‘biota-environment') analysis (Clarke, 1993) was applied to investigate associations between environmental variables to biotic community composition using Spearman's rank correlation method. Briefly, in the current example, BIOENV searches over subsets of the environmental variables for a combination that provides the best explanatory variables between meiofaunal community assemblages and environmental variables. Fourteen potential environmental drivers were included for both estuaries: spring tidal range, mean velocity, maximum velocity, mean bed shear stress, maximum bed shear stress, D50, D10, % clay, % silt, % fine sand, % medium sand, % coarse sand, % gravel and salinity range. In the Thames estuary, macrofauna species richness, abundance and biomass data were available for all sites except Cadogan Pier (CP), Kew (K) and London Bridge (LB). Therefore, for the Thames estuary, the BIOENV procedure was performed on the 20 sampling sites without the macrofauna data, and on 17 sites (after exclusion of CP, K and LB sites) with the macrofauna data. In the Mersey estuary, because of the very low abundance (three or less animals recorded at five sites: TN, EG, RF, EF and SK) or absence (at six sites: EB, LA, HH, CM, FF and FW) of macrofauna species, in particular at sites with large mobile sand waves consisting of coarse sand, macrofauna species richness, abundance and biomass were not included in the BIOENV analyses. To complement the BIOENV analyses, we also performed a canonical correspondence analysis (CCA) in the package ‘vegan' in R (Oksanen *et al.,* 2013) to further test and visualise the relationships between the environmental variables and community composition. Because correlated explanatory variables can make the interpretation of CCA outputs difficult, explanatory variables that had a correlation coefficient of >0.7 were excluded from the analysis. After this selection, only mean tidal velocity, the sediment D50 and salinity range data were retained and the best fitting model was selected by Akaike information criterion using a stepwise algorithm.

In order to investigate the geographical contribution to the marine component of estuarine biodiversity, we subsampled UK-only sites from the recent littoral beach data presented in Fonseca *et al.* (2014) and performed presence/absence Sørensen Index Mantel tests in PRIMER v6 to explore isolation by closest coastal distance effects on community composition.

Finally, we used partial least squares (PLS) regressions to assess which environmental variables were the most important drivers of meiofaunal diversity, with the PLS package in R (Mevik and Wehrens, 2007). The PLS regressions have been developed to deal with cases where there are many explanatory variables in relation to the number of observations and/or with cases of severe multicollinearity (Carrascal *et al.,* 2009) and are therefore particularly suited for this analysis. A PLS regression is a linear regression of one or more response variables onto a number of components called latent variables. The latent variables are linear combinations of the factors, also called predictor variables. They are constructed so that the original multicollinearity is reduced to a lower number of orthogonal factors. The variable importance in projection (VIP) approach (Chong and Jun, 2005) was used to order the pertinent original explanatory variables by rank importance, that is, response variables with VIP values >1 were considered pertinent. Each PLS analysis generates the VIP values for each response variable, as well as the variance (*R*^2^) explained by each of the two components. This analysis was performed within each estuary for the meiofaunal phyla (whole data set and per phylum), protists (Alveolata and stramenopiles) and fungi.

## Results

The raw sequencing data were analysed in three ways: (1) Octupus, non-rarefied, NCBI annotated; (2) Octupus, rarefied to the lowest number of reads, NCBI annotated and (3) FlowClus/QIIME, non-rarefied, Silva annotated. The diversity patterns and overlying conclusions were unaffected by the different bioinformatic workflows, and hence below we present the results obtained with FlowClus/QIIME pipeline.

**Figure 2:**
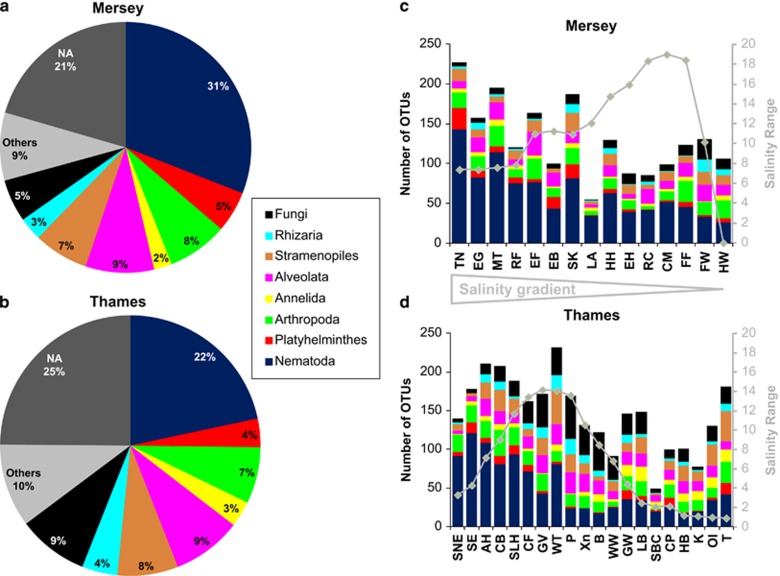
(**a**, **b**) Summary of OTU pie charts for the Thames and Mersey estuaries. (**c**, **d**) Stacked histograms showing taxonomy composition for the Thames and Mersey estuaries. At each site, three replicates were pooled. Others include Mollusca, Gastotricha, Tardigrada, Kinorhyncha, Rotifera, Viridiplantae, Porifera, Cnidaria, Bryozoa, Brachiopoda, Rhodophyta, Entoprocta, Craniata, Urochordata, Nemerta, Cryptophyta, Apusozoa, Cryptista, Amoebozoa and Holozoa. NA, not assigned. See Supplementary Table S1 for site abbreviations.

The 454 Roche sequencing yielded 957 216 reads, with each sample exhibiting between 1044 and 30 786 sequence reads (Supplementary Table S2). The numbers of reads for the combined data set were dominated by Nematoda (55.38%) and Arthropoda (18.48%) (Supplementary Table S3). For the Thames and Mersey estuaries, 1496 and 1131 OTUs were recovered respectively (Supplementary Table S3). OTU richness was dominated by Nematoda (493 OTUs across both estuaries, representing 22% of the OTUs in Thames and 31% in Mersey), followed by substantial contributions by Alveolata (173 OTUs), stramenopiles (146 OTUs), Arthropoda (138 OTUs), Platyhelminthes (89 OTUs) and a range of additional taxa from up to 27 separate phyla (Figure 2 and Supplementary Table S3). An analysis of variance also revealed no significant difference in OTU richness between 454 Roche plate gaskets (F=2.24, *P*=0.088).

Samples were taken over a distance of ∼46 km from Mersey and 106 km from Thames, representing the full spectrum of the salinity gradient in each ecosystem. The spring tidal range ranged from 1.43 to 6.56 m in Thames and between 0 and 8.64 m in Mersey, resulting in maximum flow velocities of 0.68–2.07 and 0–1.70 m s^−1^ and salinity ranges of 0.89–14.16 and 0–18.97 p.p.t. respectively (Supplementary Tables S4 and S5).

A *t*-test revealed that richness between the two estuaries was not significantly different for Nematoda (*t*_33_=−1.107, *P*=0.276), Platyhelminthes (*t*_33_=−0.746, *P*=0.461), Arthropoda (*t*_33_=0.555, *P*=0.582), Alveolata (*t*_33_=0.326, *P*=0.747) and Rhizaria (*t*_33_=1.518, *P*=0.139). However the Annelida (*t*_33_=3.014, *P*=0.006), the stramenopiles (*t*_33_=2.350, *P*=0.026) and Fungi (*t*_33_=4.009, *P*=0.000) were all significantly richer in Thames as compared with Mersey. Total richness was higher throughout the Thames samples, with only 20% of sampling stations yielding 100 or less OTUs as compared with 33.3% of the Mersey samples. The overall distributions of OTU richness were very different between the two estuaries. Thames exhibited a peak of richness in the euhaline zone and an increase in freshwater richness following reduced diversity throughout the meso- and oligohaline zones. Conversely, Mersey exhibited a general trend of reduced diversity from the marine, through to the freshwater and the oligohaline zone (Figure 2).

**Figure 3:**
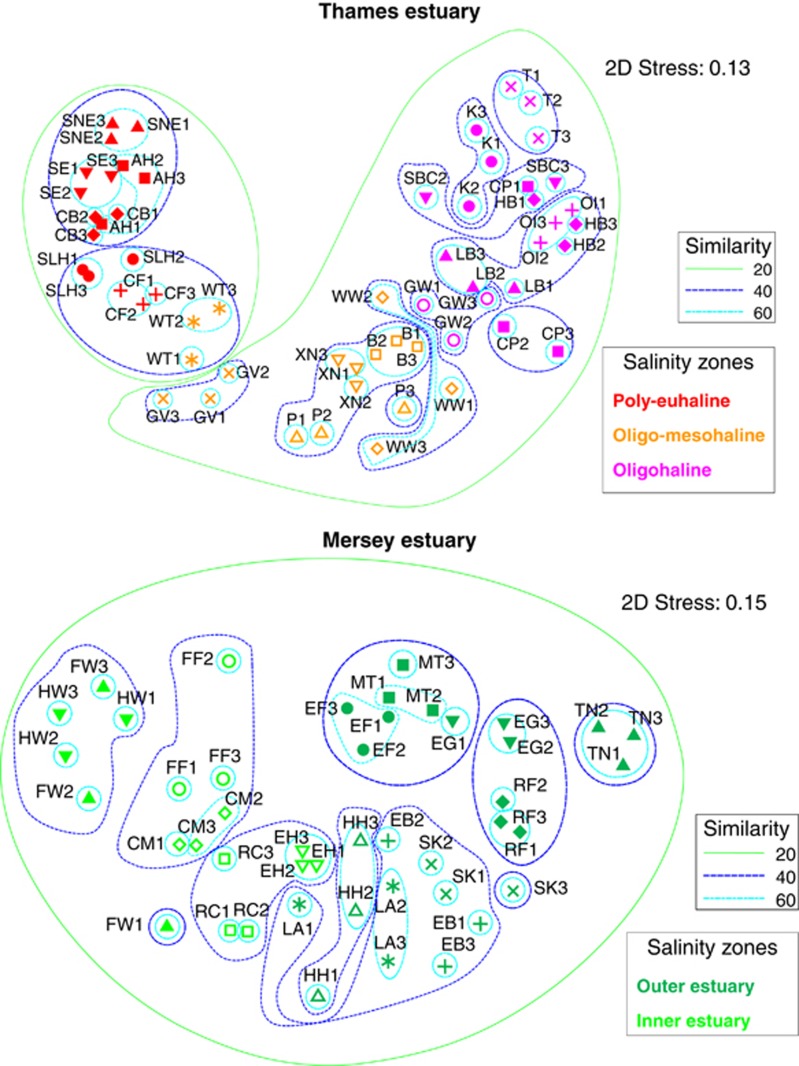
Multidimensional scaling (MDS) ordination for taxonomic patterns of meiofaunal communities based on Sørensen similarities of OTU presence/absence data for the Thames and Mersey estuaries analysed separately. Community-based similarity contours are shown (20%, 40%, and 60%). See Supplementary Table S1 for site abbreviations.

**Figure 4:**
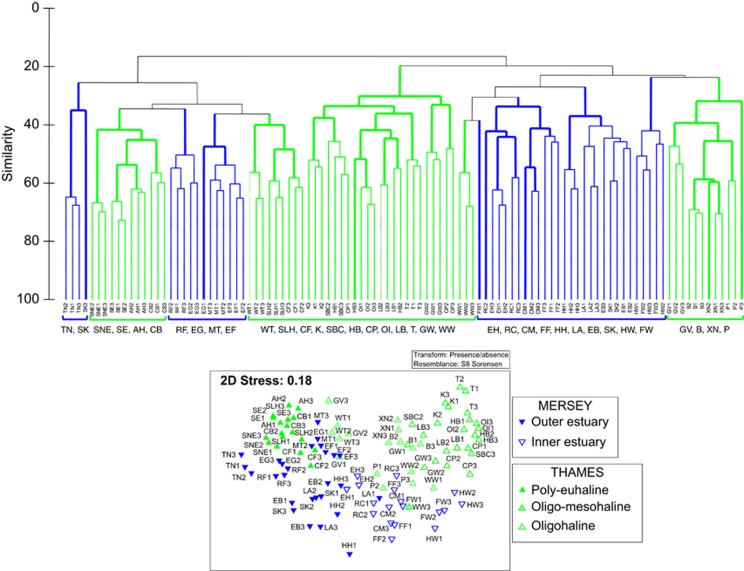
Cluster analysis and multidimensional scaling (MDS) ordination for taxonomic patterns of meiofaunal communities based on Sørensen similarities of OTU presence/absence data for the combined estuaries. In the dendrogram, thick lines represent samples that are significantly differentiated based on a SIMPROF (‘similarity profile' permutation tests) analysis. The two estuaries have been colour coded (M, Mersey in blue; T, Thames in green). See Supplementary Table S1 for site abbreviations.

In general, the replicates within stations clustered together for both estuaries (Supplementary Figures S1 and S2). In Thames, SIMPROF analyses recovered discrete poly-euhaline, oligo-mesohaline and oligohaline communities at 25% similarity (Supplementary Figure S1). In Mersey, two communities were discretely discriminated at 25% similarity, separated by the geomorphology of the river basin, delineated by a discontinuity between the inner and outer estuaries (separated between Hale Head and East Hale) (Supplementary Figure S2). In the multidimensional scaling ordinations, community composition of each estuary followed a continuum from the freshwater to marine sides (Figure 3). In the combined multidimensional scaling plots of both estuaries, the communities derived from the broad oligohaline, oligo-mesohaline and poly-euhaline zones in the two estuaries clustered separately in the SIMPROF analyses (Figure 4).

Moving beyond the phylum level and focusing on the Nematoda (the most abundant phylum in terms of number of reads and OTUs), further trends can be explored within the data. In the Thames estuary, four families are abundant in the poly-euhaline zone, Chromadoridae, Comesomatidae, Desmodoridae and Xyalidae, whereas in the oligohaline zone Tripylidae is the most abundant Nematoda family (Supplementary Figure S3). In the outer part of the Mersey estuary (sites The Narrows to Speke), seven families represent the most number of reads: Axonolaimidae, Chromadoridae, Comesomatidae, Desmodoridae, Sphaerolaimidae, Xyalidae and Thoracostomopsidae. In the inner part of the Mersey estuary (sites Liverpool Airport to Howley Weir), the family Xyalidae is overwhelmingly dominant, whereas in the two extreme freshwater sites (Forest Way and Howley Weir) the Tripylidae and Monhysteridae additionally feature (Supplementary Figure S4).

**Figure 5:**
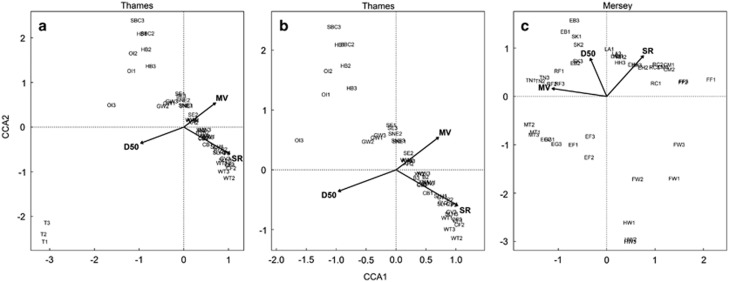
Canonical correspondence analysis plots (first two axes, CCA1 and CCA2) for (**a**) the Thames estuary, total sites; (**b**) the Thames estuary, total sites minus Teddington (that forces strong ordination because of granulometry effects); and (**c**) the Mersey estuary. Arrows indicate direction of the gradient according to the specified variable of D50 (particle diameter at 50% in the cumulative distribution of grain sizes), mean velocity (MV) and salinity range (SR).

Considering meiofaunal community assemblages, the BIOENV analysis showed that both sediment granulometry and mean salinity range optimally explained differences in community composition of the sampled meiofauna in both estuaries (Tables 1 and 2), with additional effects of hydrodynamic flow in Mersey (Table 1) and macrofauna species richness in Thames (Table 2b). Similarly, Figure 5 summarises 74% and 78% of the total constrained inertia of the final selected models of the CCA analyses, with all three retained environmental variables showing highly significant associations with community composition in Mersey (all *P*=0.01) and Thames (all *P*=0.005), respectively. The Mantel tests performed on the Sørensen Index community similarity measures from the UK-only sites from Fonseca *et al.* (2014) showed no association with geographic distance.

For the PLS regression analyses, environmental characters were ranked according to the strongest association between dependent and predictive variables, regression plots assessed for spurious associations and outlier effects. Data are presented only for phyla with 10 OTUs in total or more. In Mersey, there were few clear associations with the environmental predictive variables. However, of these, there were strong positive associations between hydrodynamic flow and total biodiversity, Nematoda and Annelida richness, and between small sediment granulometry (D10) and Platyhelminthes richness. There was also a negative association between hydrodynamic flow and Fungi richness (Table 3). Conversely, in Thames, all of the hydrodynamic associations between the predictor variables and biodiversity (all phyla, Nematoda and Arthropoda) exhibited a negative association. There was also a negative association between salinity range and Mollusca richness. Positive associations were observed between granulometry and Annelida and Mollusca richness. Additional positive associations were observed between Nematoda richness and macrofauna species richness and biomass, and between salinity range and the richness of Fungi/Rhizaria (Table 4).

## Discussion

### The metabarcoding approach and ecosystem diversity

A number of recent reviews and studies have highlighted the advantages of analysing environmentally sourced community DNA as compared with using traditional taxonomical or ecological approaches (Baird and Hajibabaei, 2012; Taberlet *et al.,* 2012a, 2012b), and the present study supports this view. Moreover, here we used community extractions and longer PCR amplicons (ca. 450 bp) to preferentially amplify DNA from living communities, as opposed to environmental DNA (Bohmann *et al.,* 2014), that generally cannot be amplified from dead organisms, or degraded environmental DNA using longer PCR amplicons (Valentini *et al.,* 2009). The use of a replicated sample design here shows that replicate samples within a station were generally not statistically different from each other, with the exception of SK, EG, RC, CM and FW in Mersey and P, XN, CP, LB and HB in Thames (see Figure 3 and Supplementary Figures S1 and S2). However, in all these examples, clustering represented geographically proximate sampled sites, likely representing ecologically distinct habitats.

Notably, and as for Fonseca *et al.* (2010), recovered estimates of Nematoda richness, using the QIIME pipeline at a 96% cutoff identity, were similar to those derived from traditional taxonomy for the Thames estuary (Attrill, 1998; Ferrero *et al.,* 2008) (for example, 209 spp. identified morphologically at 8 sites by Ferrero *et al.,* 2008; 324 OTUs found at 20 sites (Supplementary Table S3 this study); 199 OTUs found when restricting the molecular data to the same 8 sites than Ferrero *et al.,* 2008). Moreover, the metabarcoding data yielded the same ecological pattern of community turnover along the salinity gradient as traditional analyses based solely on nematode diversity (Ferrero *et al.,* 2008) (Supplementary Figures S5 and S6). Comparisons of the ecological signal between metabarcoding studies and traditional ecological surveys (Ji *et al.,* 2013) will become an increasingly important consideration by environmental agencies regarding the uptake of second-generation sequencing approaches to perform routine biomonitoring studies (Baird and Hajibabaei, 2012).

### Inter-ecosystem variability in richness and composition

The first stark difference between the Thames and Mersey ecosystems was that sufficient macroinvertebrate taxa were manually recovered for hypothesis testing only in the Thames estuary. The second difference concerned the salinity-aligned biodiversity profiles of the two ecosystems (Figure 2). Whereas Thames exhibited a sinusoidal relationship between taxon richness and salinity range, extending from the marine euhaline, poly-mesohaline to the oligohaline zone, the biodiversity in Mersey steadily declined from the marine environment into the freshwater sites. Ecoclines represent gradual ecological change over an environmental gradient between two systems (for example, altitudinal/salinity gradients) (Attrill and Rundle, 2002). Therefore, both Thames and the Mersey adhere to an ecocline model of biodiversity *composition* and, interestingly, also taxonomy, because their meiofaunal distribution and composition vary according to the salinity gradient (Figure 2 and Supplementary Figures S3 and S4). Such patterns in β-diversity are predicted by ecological theory and morphological classification of nematode diversity within estuarine ecosystems (Giere, 2009). In contrast, an ecotone is an area of rapid ecological change between two different and relatively homogeneous communities. In this respect, Thames, with its peak of richness in the intermediate poly-euhaline zone (sites Allhallows and Cavney Island; Figure 2), adheres to the ecotone model of *richness* proposed recently in Whitfield *et al.* (2012). Indeed, this intermediate peak of richness may be the result of contributions of species diversity derived from the mixing of the oligo-mesohaline (for example, site WT) and poly-euhaline (for example, sites SNE, SE, SLH and CF) communities (Figure 3).

From a biomonitoring perspective, it is interesting that the communities within the different salinity zones clustered by estuary (notably so in the freshwater zones; Figure 4), suggesting that components of the community composition of each estuary are ecosystem specific. Considering selection, drift, speciation and dispersal as the four processes that likely influence community composition (Vellend, 2010), either selection by the environment or dispersal limitation are likely to be attributable to the observed differences in community structure. Mantel tests were unable to detect isolation-by-distance relationships of the UK-only meiofauna data presented in Fonseca *et al.* (2014), suggesting that dispersal limitations were unlikely to have caused such relationships for the marine communities. Nevertheless, dispersal may be further impeded by the land–sea interface for freshwater communities, although notable differences between Mersey and Thames freshwater sites were likely to be driven by sediment granulometry habitat effects (Figure 5) (Giere, 2009). Considering selection, the Thames estuary has experienced historical bouts of extreme pollution, predominantly as a consequence of unregulated releases of sewage that caused serious depletion of oxygen levels (Tinsley, 1998); improvements in water quality are reflected by an increase in piscivorous birds (Attrill, 1998) and a resident seal population (Attrill, 1998). Although water quality in Mersey is continually improving because of initiatives set up in recent years under the Mersey Basin Campaign (Struthers, 1997) and changes in industrial practices, high levels of organic (for example, polyaromatic hydrocarbons and polychlorinated biphenyls) and inorganic (for example, mercury, zinc and chromium) contaminants still persist in its sediment habitats (Langston *et al.,* 2006). Unfortunately, comparative ecotoxicological data for the studied sites at the time of sampling are not available and hence further investigations are precluded here. Nevertheless, ecosystem-specific factors not measured here are likely to influence community similarity patterns (for example, ecotoxicology and geology) and may offer biomonitoring potential for the characterisation of ecosystem health or condition. Companion analyses across geographically and ecologically disparate ecosystems with differing levels of ecotoxicological exposures will further disentangle the relationships between selection and dispersal in relation to estuarine microbial biogeography (Martiny *et al.,* 2006; Vellend, 2010).

### Heterogeneous drivers of microbial eukaryote biodiversity

Following twenty-first century reassessments of the relevance of the Remane model of estuarine biodiversity (Whitfield *et al.,* 2012), substantial focus has been placed on the role of salinity stress (that is, range) in shaping patterns of estuarine biodiversity (Attrill, 2002; Whitfield *et al.,* 2012). Nevertheless, the abiotic environment is complex with hydrological, sediment granulometry and macrofaunal ‘top-down' components, where salinity is just one factor. Regarding the BIOENV analysis of β-diversity, salinity range along with other factors (hydrodynamics and granulometry in Mersey and macrofaunal diversity and granulometry in Thames) were shown to be the main factors explaining meiofaunal community distribution (Tables 1 and 2). Similarly, salinity range, hydrodynamics and granulometry were all significantly associated with community turnover in the CCA analyses. A substantial shift in community composition according to an increase in granulometry size in the freshwater Teddington site, accompanied by a strong effect of salinity range, can be seen in Thames. Conversely, all three environmental factors were associated with community separation throughout both axes of the Mersey CCA plots (Figure 5). However, considering the PLS regression analyses of α-diversity, salinity range was identified as having a significant negative association with Mollusca and positive association with fungal and Rhizaria richness only in Thames (Table 4). Therefore, assessed in isolation, salinity can appear to be a relevant driver of richness, but additional environmental drivers may be more important and may go unnoticed with limited hypothesis testing. Similarly, authors have proposed models of estuarine biodiversity in relation to salinity (Attrill, 2002; Whitfield *et al.,* 2012), but the comparisons between the Thames and Mersey ecosystems here exhibit substantial differences in composition (including shared and unique taxa to each ecosystem), richness and associated drivers of biodiversity, highlighting the need for further comparative studies and potential identification of ecologically representative taxa (Chariton *et al.,* 2010).

Considering the top three meiofaunal phyla, the prevalence of the Nematoda and Platyhelminthes OTUs seen in the present data have been observed in previous metabarcoding analyses of marine meiofaunal biodiversity (Fonseca *et al.,* 2010, 2014), although the Arthropoda in the present analyses feature as the second most dominant phylum (Nematoda>Arthropoda>Platyhelminthes OTUs). Despite both having a well-adapted *vermiform* body for interstitial life (Giere, 2009), Nematoda and Platyhelminthes responded to different biotic and abiotic drivers of richness. In Mersey, sediment granulometry was the most significant factor affecting Platyhelminthes richness (Table 3), whereas hydrodynamics in Mersey (Table 3) and metrics associated with hydrodynamics and macrofaunal bioturbation in Thames (Table 4) showed the most significant associations with Nematoda richness. The positive associations with flatworm richness could reflect a larger body size of turbellarians sampled in the study, accompanied by increased habitat diversity, and/or space associated with larger sediment particles. The Nematoda in Thames likely benefitted from the multiple side effects of the top-down processes of macrofaunal diversity and bioturbation (for example, food, aeration, secondary production, microbial activity and so on) (Branch and Pringle, 1987). Again, the comparisons between OTU richness and the various parameters of hydrodynamic flows that we have modelled show that biodiversity trends in Thames and Mersey are diametrically opposed in relation to flow. For total richness, Nematoda and Annelida, there were positive associations with flow in Mersey, but negative associations for total richness, Nematoda and Arthropoda in Thames, respectively (Tables 3 and 4), suggesting a depletion of biodiversity in areas of slow-flowing waters in Mersey. Therefore, either water quality or low oxygen levels in Mersey may have caused a reduction in microbial eukaryote biodiversity immediately before sampling, notably highlighted by significantly higher numbers of stramenopiles and Arthropoda OTUs in the Thames. Indeed, the geomorphology of Mersey presents a challenge for the exchange of water in and out of the ecosystem (NRA 1995), via the retention of water and increased residence times. Conversely, Thames has experienced extensive bouts of canalisation and hydrodynamic measures that will increase flows and decrease water residence times over short- and long-term tidal cycles (Thames Estuary Partnership, personal communication).

### Opportunities and limitations of environmental metabarcoding approaches

The present study showcases the scalable, objective and cost-effective benefits of metabarcoding compared with traditional taxonomy approaches. From a quantitative perspective, the nSSU gene exhibits pronounced interspecific variation in copy number (Bik *et al.,* 2013). Accordingly, interspecific biodiversity metrics represent nSSU diversity, and not species diversity. Nevertheless, intraspecific nSSU comparative measures are predicted to reflect multicellular abundance, as for bacterial (cellular) abundance using 16S ribosomal RNA. In addition, typical second-generation sequencing nSSU taxonomy gene loci do not resolve species in all cases (Creer *et al.,* 2010). Nevertheless, by working in conjunction with taxonomists, molecular ecologists can investigate reverse taxonomy approaches (Creer *et al.,* 2010) to forge the necessary links between metabarcoding data sets and morphology/functional ecology. In the future, the use of rapidly evolving second-generation sequencing platforms and the incorporation of further environmental factors such as nutrients, physicochemical and ecotoxicological data will only enhance this analytical power with the aim of constructing ecosystem scale models in order to understand not only baseline diversity of all kingdoms of life, but also community responses to further environmental stressors and change.

## Figures and Tables

**Table 1:** Summary of results from the biota-environment (BIOENV) analysis in the Mersey estuary showing the 10 best combinations of environmental variables associated with the highest correlation between the meiofaunal and environmental data matrices

*No. of variables*	*Correlation*	*Environmental variables*
4	0.728	Spring tidal range, mean velocity, peak velocity, mean salinity range
5	0.726	Spring tidal range, mean velocity, peak velocity, % silt, mean salinity range
5	0.721	Spring tidal range, mean velocity, peak velocity, % clay, mean salinity range
6	0.718	Spring tidal range, mean velocity, peak velocity, mean bed shear stress, % clay, mean salinity range
3	0.716	Spring tidal range, mean velocity, mean salinity range
6	0.716	Spring tidal range, mean velocity, peak velocity, mean bed shear stress, % silt, mean salinity range
3	0.716	Spring tidal range, mean velocity, peak velocity
5	0.714	Spring tidal range, mean velocity, peak velocity, mean bed shear stress, mean salinity range
5	0.714	Spring tidal range, mean velocity, mean bed shear stress, % clay, mean salinity range
4	0.713	Spring tidal range, mean velocity, peak bed shear stress, mean salinity range

Correlation values correspond to Spearman's rank correlation coefficient (ρ).

**Table 2:** Summary of results from the biota-environment (BIOENV) analysis in the Thames estuary

*No. of variables*	*Correlation*	*Environmental variables*
*(a)*		
3	0.689	D10, % fine sand, mean salinity range
2	0.672	% Fine sand, mean salinity range
2	0.667	D10, % fine sand
4	0.665	Peak velocity, D10, % fine sand, mean salinity range
3	0.659	% Fine sand, % coarse sand, mean salinity range
4	0.659	Peak bed shear stress, D10, % fine sand, mean salinity range
4	0.655	D10, % fine sand, % medium sand, mean salinity range
5	0.654	Peak velocity, D10, % fine sand, % medium sand, mean salinity range
5	0.649	D10, % fine sand, % medium sand, % gravel, mean salinity range
6	0.648	Peak velocity, D10, % fine sand, % medium sand, % gravel, mean salinity range
		
*(b)*		
4	0.702	D10, % fine sand, mean salinity range, macrofauna species richness
5	0.697	D10, % fine sand, % medium sand, mean salinity range, macrofauna species richness
6	0.685	Peak velocity, D10, % fine sand, % medium sand, mean salinity range, macrofauna species richness
6	0.683	D10, % fine sand, % medium sand, % coarse sand, mean salinity range, macrofauna species richness
5	0.680	Peak velocity, D10, % fine sand, mean salinity range, macrofauna species richness
5	0.677	D10, % fine sand, % coarse sand, mean salinity range, macrofauna species richness
5	0.676	D50, % clay, % fine sand, % medium sand, mean salinity range, macrofauna species richness
3	0.676	D10, % fine sand, mean salinity range
6	0.675	Peak bed shear stress, D10, % fine sand, % medium sand, mean salinity range, macrofauna species richness
5	0.675	Peak bed shear stress, D10, % fine sand, mean salinity range, macrofauna species richness

Abbreviation: D10 and D50, particle diameter at 10% and 50% in the cumulative distribution of grain sizes.

(*a*) All stations included, macrofauna data not included. (*b*) Cadogan Pier (CP), Kew (K) and London Bridge (LB) sites excluded, macrofauna data included.

Correlation values correspond to Spearman's rank correlation coefficient (ρ).

**Table 3:** Partial least square regression results for the Mersey estuary

	*Total*	*Annelida*	*Nematoda*	*Platyhelminthes*	*Fungi*
Spring tidal range	0.85	0.47	0.98	1.08	1.51 (R^2^=0.47**)
Mean velocity	1.24 (*R*^2^=0.37*)	0.92	1.32 (*R*^2^=0.6**)	1.22	1.41 (R^2^=0.41*)
Peak velocity	1.12 (*R*^2^=0.3*)	0.86	1.28 (*R*^2^=0.56**)	1.02	1.46 (R^2^=0.44**)
Mean bed shear stress	1.32 (*R*^2^=0.42**)	1.1 (R^2^=0.35*)	1.35 (*R*^2^=0.62***)	1.14	1.18 (R^2^=0.29*)
Peak bed shear stress	1.25 (*R*^2^=0.38*)	1.08 (*R*^2^=0.34*)	1.33 (*R*^2^=0.6**)	1.05	1.22 (R^2^=0.31*)
D50	0.21	0.69	0.04	1.12	0.88
D10	0.1	0.57	0.14	1.25 *(R*^2^=0.27*)	0.87
Salinity range	0.75	0.59	0.48	0.81	0.11
Macrofauna SR	1	1.49	0.85	0.46	0.17
Macrofauna biomass	1.38	1.22	1.29	1.1	0.34
Macrofauna abundance	0.82	1.4	0.67	0.19	0.07

Abbreviations: D10 and D50, particle diameter at 10% and 50% in the cumulative distribution of grain sizes; SR, species richness.

Data are presented only for phyla with 10 operational taxonomic units (OTUs) in total or more. Above are reported the importance score (variable importance in projection (VIP)) for the first latent variable (see Materials and methods). Positive associations are underlined and negative associations are shown in italics. In brackets are *R*^2^ values as well as significance level: *P<0.05; **P<0.01; ***P<0.001.

**Table 4:** Partial least square regression results for the Thames estuary

	*Total*	*Annelida*	*Nematoda*	*Arthropoda*	*Fungi*	*Mollusca*	*Rhizaria*
Spring tidal range	1.03	0.7	1.22	1.35 (R^2^=0.4, *)	1.36	0.4	0.76
Mean velocity	0.89	0.35	0.82	0.77	0.64	0.74	0.52
Peak velocity	1.31 (R^2^=0.47**)	1.03	1.31 (R^2^=0.61**)	1.41 (R^2^=0.44*)	0.3	1.04	0.16
Mean bed shear stress	1	0.52	0.9	0.9	0.47	0.4	0.33
Peak bed shear stress	1.35 (R^2^=0.5**)	0.97	1.26 (R^2^=0.56**)	1.4 (R^2^=0.43*)	0.04	1.06	0.37
D50	0.78	1.43	0.73	0.68	0.14	0.18	0.02
D10	1.07	1.86 (*R*^2^=0.54**)	0.94	1.04	0.8	1.74 (*R*^2^=0.38*)	1.01
Salinity range	1.01	1.18	0.43	0.78	1.89 (*R*^2^=0.52**)	1.95 (R^2^=0.48**)	2.57 (*R*^2^=0.48**)
Macrofauna SR	1.04	0.17	1.16 (*R*^2^=0.47*)	0.96	1.33	0.54	0.89
Macrofauna biomass	0.6	1.01	1.13 (*R*^2^=0.45*)	0.79	1.28	0.05	1.13
Macrofauna abundance	0.65	0.41	0.71	0.36	0.9	0.87	0.41

Abbreviations: D10 and D50, particle diameter at 10% and 50% in the cumulative distribution of grain sizes; SR, species richness.

Data are presented only for phyla with 10 operational taxonomic units (OTUs) in total or more. Above are reported the importance score (variable importance in projection (VIP)) for the first latent variable (see Material and methods). Positive associations are underlined and negative associations are shown in italics. In brackets are *R*^2^ values as well as significance level: *P<0.05; **P<0.01; ***P<0.001.
