# Supplementary material for: Environmental metabarcoding reveals heterogeneous drivers of microbial eukaryote diversity in contrasting estuarine ecosystems
Source: ISME J. 2014 Nov 25;9(5):1208–21. doi: 10.1038/ismej.2014.213 (PMC4409164; doi:10.1038/ismej.2014.213)

Table S1. Sampling sites locations.

| **Estuary** | **Site Name** | **Label** | **Sampling date** | **Latitude** | **Longitude** |
| --- | --- | --- | --- | --- | --- |
| Thames | Shoebury Ness | SNE | 18/06/2008 | 51°31'40.32"N | 0°48' 43.62"E |
|  | Southend-on-Sea | SE | 18/06/2008 | 51°31'46.44"N | 0°43' 10.62"E |
|  | Allhallows | AH | 21/06/2008 | 51°28'52.56"N | 0°38'47.58"E |
|  | Cavney Island | CB | 18/06/2008 | 51°30'41.10"N | 0°35'40.32"E |
|  | Stanford Le Hope | SLH | 18/06/2008 | 51°30'20.76"N | 0°27'27.30"E |
|  | Coalhouse Fort | CF | 18/06/2008 | 51°27'35.52"N | 0°25'54.06"E |
|  | Gravesend | GV | 21/06/2008 | 51°26'43.26"N | 0°22'12.12"E |
|  | West Thurrock | WT | 20/06/2008 | 51°28'04.25"N | 0°17'26.24"E |
|  | Purfleet | P | 20/06/2008 | 51°29'02.28"N | 0°13'42.06"E |
|  | Crossness | Xn | 19/06/2008 | 51°30'45.54"N | 0°07'52.62"E |
|  | Beckton | B | 20/06/2008 | 51°30'52.44"N | 0°05'29.34"E |
|  | Woolwich | WW | 21/06/2008 | 51°29'41.64"N | 0°03'04.08"E |
|  | Greenwich | GW | 20/06/2008 | 51°29'04.44"N | 0°00'19.20"W |
|  | London Bridge | LB | 20/06/2008 | 51°30'24.80"N | 0°05'10.59"W |
|  | South Bank Centre | SBC | 19/06/2008 | 51°30'25.08"N | 0°07'02.16"W |
|  | Cadogan Pier | CP | 21/06/2008 | 51°29'00.41"N | 0°09'54.41"W |
|  | Hammersmith Bridge | HB | 19/06/2008 | 51°29'15.24"N | 0°13'49.44"W |
|  | Kew | K | 20/06/2008 | 51°29'15.00"N | 0°17'13.62"W |
|  | Old Isleworth | OI | 19/06/2008 | 51°28'14.94"N | 0°19'11.76"W |
|  | Teddington | T | 19/06/2008 | 51°25'49.50"N | 0°19'18.30"W |
| Mersey | The Narrows | TN | 20/07/2008 | 53°26'32.23"N | 3°02'13.58"W |
|  | Egremont | EG | 20/07/2008 | 53°25'08.53"N | 3°01'23.12"W |
|  | Mersey Tunnel | MT | 20/07/2008 | 53°22'57.90"N | 3°00'15.42"W |
|  | Rock Ferry | RF | 20/07/2008 | 53°22'12.35"N | 2°59'40.09"W |
|  | Eastham Ferry | EF | 20/07/2008 | 53°19'58.02"N | 2°57'28.26"W |
|  | Ellesmere Bank | EB | 20/07/2008 | 53°19'17.70"N | 2°56'23.34"W |
|  | Speke | SK | 21/07/2008 | 53°20'02.75"N | 2°53'08.98"W |
|  | Liverpool Airport | LA | 21/07/2008 | 53°19'35.76"N | 2°51'35.46"W |
|  | Hale Head Shore | HH | 21/07/2008 | 53°18'39.48"N | 2°47'42.48"W |
|  | East Hale | EH | 21/07/2008 | 53°19'40.14"N | 2°46'42.96"W |
|  | Runcorn | RC | 21/07/2008 | 53°20'51.66"N | 2°44'02.88"W |
|  | Cuerdley Marsh | CM | 21/07/2008 | 53°21'20.68"N | 2°40'26.20"W |
|  | Fiddlers Ferry | FF | 21/07/2008 | 53°22'22.26"N | 2°39'40.74"W |
|  | Forest Way | FW | 23/07/2008 | 53°22'46.32"N | 2°36'57.06"W |
|  | Howley Weir | HW | 23/07/2008 | 53°23'04.56"N | 2°35'04.83"W |

Table S2. TAG used for 454 sequencing and number of reads per site.

| **Estuary** | **Site** | **Sample** | **TAG*** | **No. of reads** |
| --- | --- | --- | --- | --- |
| Thames | Shoeburyness East | SNE1 | GTGAG | 7557 |
|  |  | SNE2 | TACGC | 3219 |
|  |  | SNE3 | GTCAC | 7537 |
|  | Southend on Sea | SE1 | CTACT | 17466 |
|  |  | SE2 | TCTGT | 11959 |
|  |  | SE3 | GATGA | 8257 |
|  | Allhallows | AH1 | TACAT | 7248 |
|  |  | AH2 | GACGT | 30786 |
|  |  | AH3 | TACTA | 5931 |
|  | Canvey Island | CB1 | ACTAT | 4331 |
|  |  | CB2 | AGTGT | 3411 |
|  |  | CB3 | ATAGT | 16563 |
|  | Stanford Le Hope | SLH1 | ACGCA | 7242 |
|  |  | SLH2 | AGACA | 5197 |
|  |  | SLH3 | ATCGA | 8887 |
|  | Coalhouse Fort | CF1 | TAGCA | 16259 |
|  |  | CF2 | TGATA | 6568 |
|  |  | CF3 | TGTCA | 14262 |
|  | Gravesend | GV1 | GACTC | 3438 |
|  |  | GV2 | GAGAC | 9771 |
|  |  | GV3 | TGCAC | 7360 |
|  | West Thurrock | WT1 | GTGAG | 8258 |
|  |  | WT2 | TACGC | 6163 |
|  |  | WT3 | GTCAC | 8832 |
|  | Purfleet | P1 | CTACT | 7354 |
|  |  | P2 | TCTGT | 6714 |
|  |  | P3 | GATGA | 8300 |
|  | Crossness | XN1 | TGACT | 10917 |
|  |  | XN2 | ATGCT | 8880 |
|  |  | XN3 | TCGTC | 4337 |
|  | Beckton | B1 | TATAC | 19829 |
|  |  | B2 | ACGAC | 4664 |
|  |  | B3 | TCGCT | 8302 |
|  | Woolwich | WW1 | TACAT | 8350 |
|  |  | WW2 | GACGT | 2457 |
|  |  | WW3 | TACTA | 5622 |
|  | Greenwich | GW1 | ACTAT | 11678 |
|  |  | GW2 | AGTGT | 10438 |
|  |  | GW3 | ATAGT | 8562 |
|  | London Bridge | LB1 | ACTAT | 6625 |
|  |  | LB2 | AGTGT | 7056 |
|  |  | LB3 | ATAGT | 16547 |
|  | South Bank Centre | SBC2 | GCATA | 2170 |
|  |  | SBC3 | GTGAG | 5711 |
|  | Cadogan Pier | CP1 | TCTGT | 5478 |
|  |  | CP2 | GATGA | 5148 |
|  |  | CP3 | TGACT | 4015 |
|  | Hammersmith Bridge | HB1 | ACGAC | 3590 |
|  |  | HB2 | TCGCT | 4790 |
|  |  | HB3 | TACAT | 3877 |
|  | Kew | K1 | GACTC | 2551 |
|  |  | K2 | GAGAC | 1998 |
|  |  | K3 | TGCAC | 3184 |
|  | Old Isleworth | OI1 | ATGCT | 6139 |
|  |  | OI2 | TCGTC | 3937 |
|  |  | OI3 | TATAC | 3565 |
|  | Teddington | T1 | ACGCA | 9389 |
|  |  | T2 | AGACA | 12317 |
|  |  | T3 | ATCGA | 10457 |
| Mersey | The Narrows | TN1 | TGACT | 6780 |
|  |  | TN2 | ATGCT | 12572 |
|  |  | TN3 | TCGTC | 11963 |
|  | Egremont | EG1 | GTGAG | 7137 |
|  |  | EG2 | TACGC | 3576 |
|  |  | EG3 | GTCAC | 3120 |
|  | Mersey Tunnel | MT1 | TGATA | 24855 |
|  |  | MT2 | TGTCA | 27593 |
|  |  | MT3 | GACTC | 26136 |
|  | Rock Ferry | RF1 | TACGC | 1958 |
|  |  | RF2 | GTCAC | 2933 |
|  |  | RF3 | CTACT | 2236 |
|  | Eastham Ferry | EF1 | GAGAC | 28337 |
|  |  | EF2 | TGCAC | 14382 |
|  |  | EF3 | GCATA | 29708 |
|  | Ellesmere Bank | EB1 | ACGCA | 1044 |
|  |  | EB2 | AGACA | 1921 |
|  |  | EB3 | ATCGA | 2715 |
|  | Speke | SK1 | TACAT | 7642 |
|  |  | SK2 | GACGT | 7678 |
|  |  | SK3 | TACTA | 8894 |
|  | Liverpool Airport | LA1 | TAGCA | 1052 |
|  |  | LA2 | TGATA | 1694 |
|  |  | LA3 | TGTCA | 1387 |
|  | Hale Head Shore | HH1 | TATAC | 17348 |
|  |  | HH2 | ACGAC | 13538 |
|  |  | HH3 | TCGCT | 11823 |
|  | East Hale | EH1 | CTACT | 6286 |
|  |  | EH2 | TCTGT | 3359 |
|  |  | EH3 | GATGA | 4471 |
|  | Runcorn | RC1 | GACGT | 3149 |
|  |  | RC2 | TACTA | 2754 |
|  |  | RC3 | ACTAT | 3540 |
|  | Cuerdley Marsh | CM1 | AGACA | 14960 |
|  |  | CM2 | ATCGA | 19266 |
|  |  | CM3 | TAGCA | 27224 |
|  | Fiddler's Ferry | FF1 | AGTGT | 21924 |
|  |  | FF2 | ATAGT | 21078 |
|  |  | FF3 | ACGCA | 13427 |
|  | Forest Way | FW1 | TGACT | 5939 |
|  |  | FW2 | ATGCT | 6487 |
|  |  | FW3 | TCGTC | 6568 |
|  | Howley Weir | HW1 | TATAC | 28471 |
|  |  | HW2 | ACGAC | 9514 |
|  |  | HW3 | TCGCT | 7057 |
|  |  |  |  |  |
| **Total** |  |  |  | **957216** |

* TAG: five nucleotide tag used to differentiate multiple samples within a plate

Table S3. Number of reads per phylum; number of OTUs per phylum and per estuary at 96% identity cut-off. †Numbers in bracket correspond to the number of OTUs with a single read.

|  | **Number of reads** | **Number of OTUs** | | | |
| --- | --- | --- | --- | --- | --- |
| **Phylum** | **Combined** | **Thames** | **Mersey** | | **Combined†** |
| Nematoda | 530068 (55.38%) | 324 | 350 | 493 (73) | |
| Platyhelminthes | 16104 (1.68%) | 53 | 60 | 89 (12) | |
| Arthropoda | 176884 (18.48%) | 105 | 88 | 138 (11) | |
| Annelida | 55390 (5.79%) | 48 | 26 | 52 (4) | |
| Mollusca | 32978 (3.44%) | 26 | 10 | 28 (4) | |
| Gastrotricha | 4836 (0.50%) | 13 | 16 | 21 (5) | |
| Tardigrada | 1660 (0.17%) | 8 | 7 | 10 (0) | |
| Kinorhynca | 3 (<0.01%) | 2 | 0 | 2 (1) | |
| Rotifera | 3399 (0.35%) | 6 | 7 | 9 (2) | |
| Viridiplantae | 11937 (1.25%) | 57 | 33 | 61 (4) | |
| Porifera | 2 (<0.01%) | 1 | 0 | 1 (0) | |
| Cnidaria | 48 (<0.01%) | 3 | 3 | 4 (0) | |
| Bryozoa | 101 (0.01%) | 4 | 2 | 4 (1) | |
| Brachiopoda | 12 (<0.01%) | 1 | 0 | 1 (0) | |
| Rhodophyta | 45 (<0.01%) | 1 | 2 | 3 (1) | |
| Entoprocta | 3 (<0.01%) | 0 | 1 | 1 (0) | |
| Craniata (Chordata) | 8 (<0.01%) | 1 | 1 | 2 (1) | |
| Urochordata | 21 (<0.01%) | 2 | 1 | 2 (0) | |
| Nemerta | 18 (<0.01%) | 0 | 1 | 1 (0) | |
| Cryptophyta | 1 (<0.01%) | 0 | 1 | 1 (1) | |
| Apusozoa | 72 (<0.01%) | 5 | 1 | 6 (1) | |
| Alveolata | 21497 (2.25%) | 129 | 100 | 173 (22) | |
| Cryptista | 4 (<0.01%) | 0 | 2 | 2 (1) | |
| Rhizaria/Cercozoa | 4106 (0.43%) | 66 | 34 | 83 (13) | |
| Stramenopiles | 11504 (1.20%) | 113 | 80 | 146 (22) | |
| Amoebozoa | 113 (0.01%) | 4 | 1 | 4 (3) | |
| Holozoa | 852 (0.09%) | 21 | 10 | 24 (6) | |
| Uncultured eukaryote | 50 (<0.01%) | 3 | 1 | 4 (1) | |
| Unclassified Opisthokonta | 67 (<0.01%) | 6 | 1 | 7 (2) | |
| Unclassified Eukaryota | 9 (<0.01%) | 2 | 1 | 3 (1) | |
| Unassigned | 41566 (4.34%) | 361 | 229 | 499 (95) | |
| Fungi | 43858 (4.58%) | 131 | 62 | 147 (23) | |
|  |  |  |  |  | |
| **Total** | **957216** | **1496** | **1131** | **2021 (310)** | |

Table S4. Environmental data for the Thames estuary. See Table S1 for site abbreviations. TR: Tidal range; BSS: bed shear stress. D10 and D50: particle diameter at 10% and 50% in the cumulative distribution of grain sizes.

| **Site** | **Spring TR (m)** | **Mean velocity (m/s)** | **Peak velocity (m/s)** | **Mean BSS (N/m^2^)** | **Peak BSS (N/m^2^)** | **D50 (µm)** | **D10 (µm)** | **% Clay** | **% Silt** | **% Fine Sand** | **% Medium Sand** | **% Coarse Sand** | **% Gravel** | **Mean salinity range** |
| --- | --- | --- | --- | --- | --- | --- | --- | --- | --- | --- | --- | --- | --- | --- |
| SNE | 5.12 | 0.50 | 0.89 | 0.79 | 2.01 | 138 | 85.0 | 0.40 | 3.77 | 81.86 | 13.97 | 0.00 | 0.00 | 3.29 |
| SE | 5.19 | 0.52 | 0.92 | 0.84 | 2.15 | 116 | 66.8 | 0.77 | 6.59 | 86.08 | 6.57 | 0.00 | 0.00 | 4.28 |
| AH | 5.30 | 0.53 | 0.94 | 0.86 | 2.25 | 56 | 7.5 | 3.27 | 50.57 | 45.09 | 1.07 | 0.00 | 0.00 | 7.15 |
| CB | 5.38 | 0.52 | 0.93 | 0.82 | 2.23 | 55 | 6.6 | 5.77 | 51.09 | 42.96 | 0.19 | 0.00 | 0.00 | 9.02 |
| SLH | 5.55 | 0.68 | 1.26 | 1.42 | 4.07 | 15 | 2.5 | 7.87 | 77.82 | 14.30 | 0.01 | 0.00 | 0.00 | 11.69 |
| CF | 5.66 | 0.70 | 1.33 | 1.51 | 4.54 | 65 | 14.1 | 2.29 | 44.30 | 53.41 | 0.00 | 0.00 | 0.00 | 13.43 |
| GV | 5.82 | 0.77 | 1.53 | 1.85 | 5.98 | 46 | 3.3 | 5.48 | 53.69 | 37.03 | 3.74 | 0.05 | 0.00 | 14.16 |
| WT | 6.03 | 0.60 | 1.18 | 1.11 | 3.54 | 72 | 14.5 | 1.97 | 36.78 | 60.98 | 0.27 | 0.00 | 0.00 | 14.09 |
| P | 6.23 | 0.74 | 1.43 | 1.68 | 5.25 | 14 | 2.6 | 6.95 | 80.23 | 11.70 | 1.12 | 0.00 | 0.00 | 13.58 |
| Xn | 6.44 | 0.60 | 1.12 | 1.08 | 3.23 | 15 | 2.3 | 8.49 | 75.68 | 12.43 | 3.35 | 0.05 | 0.00 | 10.54 |
| B | 6.50 | 0.59 | 1.08 | 1.03 | 3.00 | 15 | 2.4 | 7.94 | 77.62 | 12.79 | 1.66 | 0.00 | 0.00 | 8.47 |
| WW | 6.56 | 0.62 | 1.12 | 1.14 | 3.22 | 14 | 2.4 | 8.16 | 79.52 | 10.58 | 1.73 | 0.00 | 0.00 | 6.80 |
| GW | 6.48 | 0.97 | 1.60 | 2.66 | 6.54 | 4875 | 289.0 | 0.07 | 0.07 | 4.40 | 14.12 | 9.50 | 71.85 | 4.39 |
| LB | 6.24 | 1.09 | 1.98 | 3.42 | 10.02 | 5785 | 184.7 | 0.73 | 0.73 | 10.92 | 9.53 | 9.18 | 68.92 | 2.46 |
| SBC | 6.12 | 0.96 | 2.07 | 2.82 | 11.00 | 2852 | 213.6 | 0.05 | 0.05 | 8.68 | 13.67 | 21.82 | 55.74 | 2.06 |
| CP | 5.93 | 0.71 | 1.80 | 1.65 | 8.31 | 6611 | 333.6 | 0.14 | 0.14 | 4.65 | 18.30 | 12.19 | 64.59 | 2.12 |
| HB | 5.69 | 0.63 | 1.90 | 1.41 | 9.19 | 306 | 239.4 | 0.03 | 0.03 | 6.68 | 78.91 | 2.21 | 12.13 | 1.18 |
| K | 5.61 | 0.54 | 2.04 | 1.10 | 10.64 | 7847 | 329.7 | 0.02 | 0.02 | 3.08 | 17.74 | 11.58 | 67.56 | 1.10 |
| OI | 5.55 | 0.38 | 1.27 | 0.47 | 4.13 | 307 | 177.2 | 0.06 | 0.06 | 13.94 | 76.29 | 8.26 | 1.39 | 0.97 |
| T | 1.43 | 0.04 | 0.68 | 0.06 | 1.17 | 7986 | 324 | 0.05 | 0.05 | 6.24 | 13.81 | 11.90 | 67.95 | 0.89 |

Table S4 – continued

| **Site** | **Macrofauna Species richness** | **Macrofauna abundance (no. of organisms per 883 cm^2^)** | **Macrofauna biomass**  **(g per 883 cm^2^)** |
| --- | --- | --- | --- |
| SNE | 4 | 176 | 75.156 |
| SE | 7 | 48 | 6.233 |
| AH | 9 | 76 | 2.615 |
| CB | 6 | 20 | 0.274 |
| SLH | 6 | 53 | 3.079 |
| CF | 5 | 540 | 4.731 |
| GV | 0 | 0 | 0 |
| WT | 3 | 114 | 1.374 |
| P | 0 | 0 | 0 |
| Xn | 4 | 210 | 5.976 |
| B | 2 | 175 | 0.353 |
| WW | 2 | 3 | 0.017 |
| GW | 1 | 10 | 0.008 |
| LB | no data | no data | no data |
| SBC | 2 | 5 | 0.012 |
| CP | no data | no data | no data |
| HB | 3 | 126 | 0.095 |
| K | no data | no data | no data |
| OI | 7 | 149 | 1.907 |
| T | 5 | 106 | 0.886 |

Table S5. Environmental data for Mersey estuary. See Table S1 for site abbreviations. TR: Tidal range; BSS: bed shear stress. D10 and D50: particle diameter at 10% and 50% in the cumulative distribution of grain sizes.

| **Site** | **Spring TR (m)** | **Mean velocity (m/s)** | **Peak velocity (m/s)** | **Mean BSS (N/m^2^)** | **Peak BSS (N/m^2^)** | **D50 (µm)** | **D10 (µm)** | **% Clay** | **% Silt** | **% Fine Sand** | **% Medium Sand** | **% Coarse Sand** | **% Gravel** | **Mean Salinity range** |
| --- | --- | --- | --- | --- | --- | --- | --- | --- | --- | --- | --- | --- | --- | --- |
| TN | 8.06 | 0.70 | 1.50 | 1.67 | 5.74 | 204 | 146.6 | 0.00 | 46.96 | 53.04 | 0.00 | 0.00 | 0.00 | 7.38 |
| EG | 8.09 | 0.67 | 1.44 | 1.53 | 5.27 | 99 | 13.5 | 1.82 | 21.99 | 68.73 | 7.46 | 0.00 | 0.00 | 7.38 |
| MT | 8.34 | 0.80 | 1.70 | 2.15 | 7.39 | 23 | 2.6 | 7.28 | 61.76 | 28.94 | 2.03 | 0.00 | 0.00 | 7.59 |
| RF | 8.39 | 0.72 | 1.54 | 1.76 | 6.10 | 137 | 84.8 | 0.23 | 4.11 | 82.53 | 13.13 | 0.00 | 0.00 | 7.84 |
| EF | 8.56 | 0.43 | 0.95 | 0.63 | 2.29 | 32 | 2.6 | 7.27 | 59.13 | 31.52 | 2.09 | 0.00 | 0.00 | 10.99 |
| EB | 8.58 | 0.39 | 0.87 | 0.53 | 1.93 | 506 | 342.7 | 0.00 | 0.00 | 0.00 | 70.56 | 29.44 | 0.00 | 11.25 |
| SK | 8.61 | 0.36 | 0.82 | 0.45 | 1.71 | 232 | 180.5 | 0.00 | 0.12 | 25.07 | 70.56 | 4.03 | 0.23 | 10.93 |
| LA | 8.64 | 0.36 | 0.80 | 0.44 | 1.65 | 147 | 88.6 | 0.00 | 0.46 | 76.39 | 23.15 | 0.00 | 0.00 | 12.07 |
| HH | 5.44 | 0.28 | 0.93 | 0.41 | 2.22 | 182 | 109.9 | 0.00 | 59.33 | 40.67 | 0.00 | 0.00 | 0.00 | 14.76 |
| EH | 4.65 | 0.28 | 0.93 | 0.39 | 2.22 | 114 | 69.5 | 0.47 | 6.03 | 89.13 | 4.37 | 0.00 | 0.00 | 15.93 |
| RC | 3.01 | 0.25 | 1.06 | 0.52 | 2.86 | 117 | 62.6 | 0.66 | 8.25 | 81.89 | 9.19 | 0.00 | 0.00 | 18.33 |
| CM | 0.77 | 0.02 | 0.50 | 0.02 | 0.63 | 111 | 58.4 | 0.94 | 9.71 | 83.73 | 5.63 | 0.00 | 0.00 | 18.97 |
| FF | 0.25 | 0.01 | 0.35 | 0.01 | 0.31 | 54 | 5.9 | 3.16 | 52.32 | 44.47 | 0.04 | 0.00 | 0.00 | 18.41 |
| FW | 0.16 | 0.00 | 0.23 | 0.00 | 0.14 | 37 | 3.6 | 5.22 | 67.16 | 27.62 | 0.00 | 0.00 | 0.00 | 10.16 |
| HW | 0.00 | 0.00 | 0.00 | 0.00 | 0.00 | 46 | 6.8 | 3.27 | 63.77 | 32.97 | 0.00 | 0.00 | 0.00 | 0.00 |

Figure S1. Cluster analysis for taxonomic patterns of meiofaunal communities based on Sørensen similarities of OTU presence/absence data for the Thames estuary, at a 96% similarity cut-off. Black dots show the clusters at 25% similarity cut-off, highlighting three zones based on the Venice Salinity Classification System. Moreover, there was no evidence that sample inclusion on specific plate/gasket combinations affected community composition.

Figure S2. Cluster analysis for taxonomic patterns of meiofaunal communities based on Sørensen similarities of OTU presence/absence data for the Mersey estuary, at a 96% similarity cut-off. Black dots show the clusters at 25% similarity cut-off, highlighting two zones. Moreover, there was no evidence that sample inclusion on specific plate/gasket combinations affected community composition.

Figure S3. Distribution of Nematoda families in the Thames estuary. The most abundant families are in bold.

Figure S4. Distribution of Nematoda families in the Mersey estuary. The most abundant families are in bold.

Figure S5. Multi-dimensional scaling (MDS) ordination for taxonomic patterns of meiofaunal communities in the Thames estuary a) using the Bray-Curtis similarity measure on observational data (fourth root-transformed nematode species data, Ferrero et al 2008); b) using the Sørensen similarity measure on molecular data (OTU presence/absence data, this study).


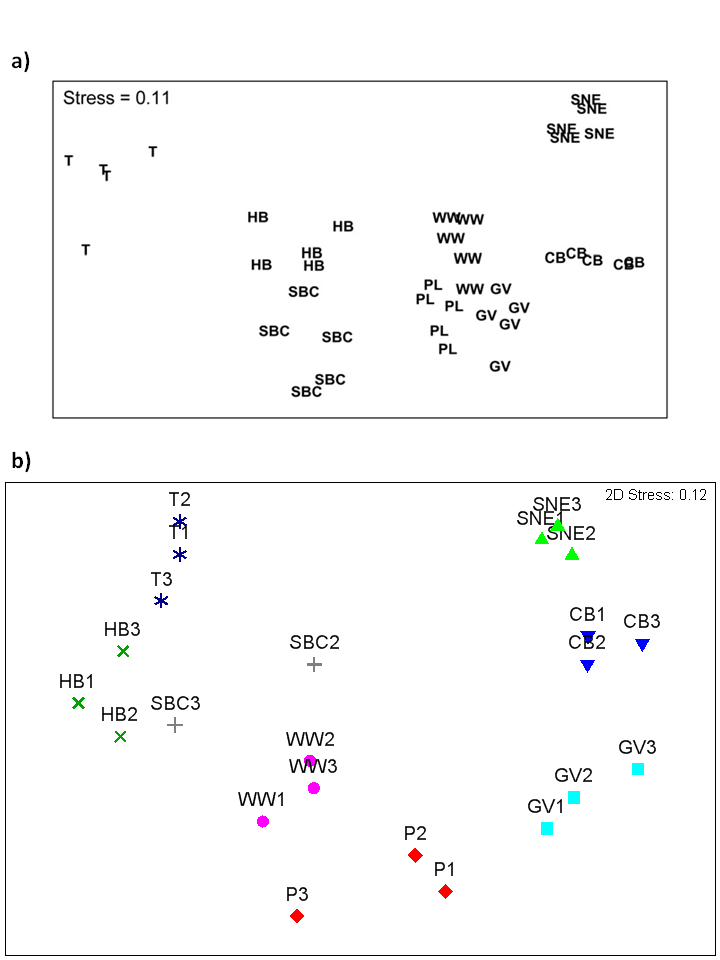


Figure S6. Kite diagram showing a) percentage density of nematode species along the Thames estuary, based on observational data (Ferrero et al 2008); b) percentage of read of nematode genus at the same sites along the Thames estuary, based on the molecular data (this study). Genus representing > 5% of the reads and present in both datasets are shown. Please note that several OTUs with the same genus annotation have been pooled for the molecular data.


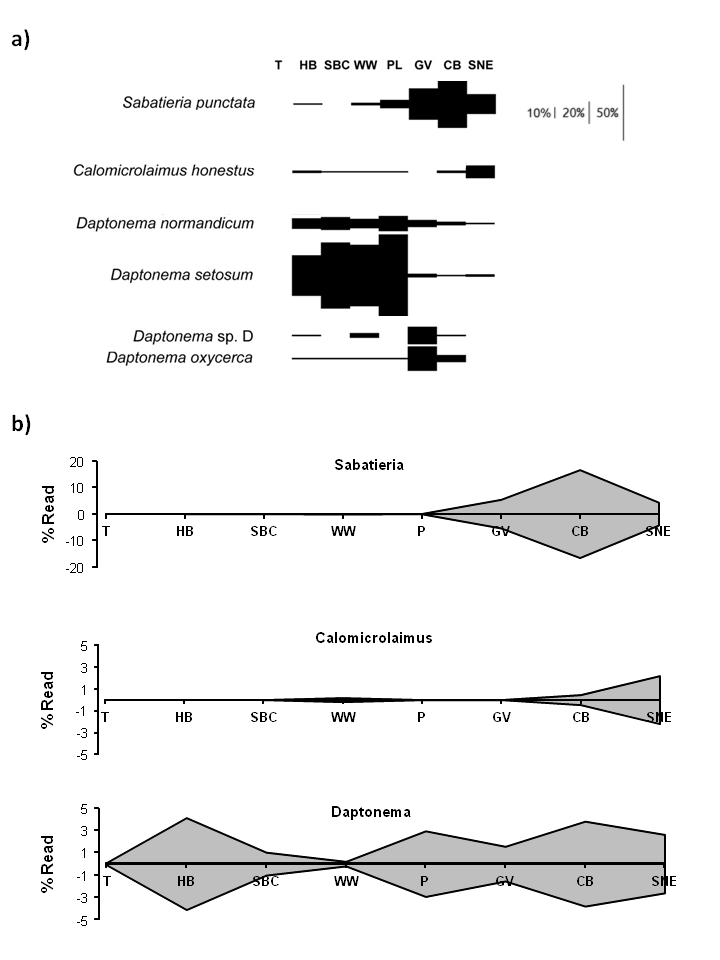

Supplement: Supplementary Table S1 [file ismej2014213x1.doc]
